# Microrheological Phenomenon and Mechanical Properties of High-Aspect-Ratio Microgroove Injection Moulding of Kaolin/PP Composites

**DOI:** 10.3390/ijms23094944

**Published:** 2022-04-29

**Authors:** Yan Lou, Xiangwei Zhou, Dongyue Zhang, Fengyu Cheng

**Affiliations:** Shenzhen Key Laboratory of High Performance Nontraditional Manufacturing, College of Mechatronics and Control Engineering, Shenzhen University, Shenzhen 518060, China; 1910293037@email.szu.edu.cn (X.Z.); 2070292097@email.szu.edu.cn (D.Z.); 2110292038@email.szu.edu.cn (F.C.)

**Keywords:** kaolin/pp composites, microgroove injection, high aspect ratio, microrheological phenomenon, mechanical properties

## Abstract

The microrheological phenomenon of kaolin-filled polypropylene (kaolin/PP) composites was investigated for the first time. The microviscosity of kaolin/PP composites was studied by changing the melt temperature and shear rate. Then, injection moulding experiments of rectangular microgrooves with different aspect ratios using kaolin/PP composites and mechanical property tests of the samples were carried out. The results showed that with increasing kaolin content, the microviscosity of the kaolin/PP composites gradually increases. The shear rate had the greatest influence on the microviscosity, and the kaolin content had the least influence. When the aspect ratio of rectangular microgrooves is small, with an increasing kaolin content, the microgroove filling rate increases, and the microstructured sample geometric shape replication effect is good; however, when the aspect ratio reaches 10:1, the microgroove filling rate decreases with an increasing kaolin content. The microstructured sample geometric shape replication effect is also poor, and size effects appear. Different factors control the microrheological morphology of composites with different aspect ratios, including the shear deformation and viscous flow of composites. The increase in kaolin content leads to a decrease in the friction coefficient and an increase in the wear resistance of the composites. We concluded that the best composite formulation for kaolin/PP composites in microinjection is the 7KL/PP composite with 7% kaolin. When the aspect ratio is 5:1, the reproduction of the microstructured sample geometry is the best, and the comprehensive mechanical properties of the sample are the best.

## 1. Introduction

Microelectromechanical systems (MEMS) have a wide range of application prospects in the national economy and military systems. Microinjection is widely used in the manufacture of microstructured parts due to its irreplaceable advantages, such as a short production cycle, low cost, and suitability for processing various parts with complex shapes. However, due to the size effect, it is difficult to fill the microstructure during the microinjection moulding process, and ensuring the filling performance and the quality of the product is the main problem faced by microinjection moulding.

Polypropylene (PP) is a typical commercial polymer with a high heat distortion temperature, good moulding fluidity, thermal stability, certain flame retardancy, and excellent mechanical properties. PP has been widely used in the electrical equipment, automotive, and defence industries and has attracted the attention of engineers and researchers. To expand the application range of polymer materials, polymer matrices are generally reinforced with fillers. Typically, carbon fibres [1,2], glass fibres [3,4], graphene [5], plant fibres [6,7], natural zeolites [8], montmorillonite [9,10,11,12], kaolin [13,14], and other reinforcing fillers are compounded with polymers to improve the mechanical and thermal properties of composites.

Among all filler materials, silicates are one of the most useful reinforcement materials, showing excellent performance in enhancing strength, and they are inexpensive and economical. Kuram et al. [3] studied the hybridisation effect of adding talc to glass fibre/polycarbonate/acrylonitrile butadiene styrene (PC/ABS) composites and found that the highest impact strength was obtained with 7.5% talc and 5% glass fibre (PC/ABS + 7.5T + 5GF) composites. The addition of talc to PC/ABS composites results in a decrease in the melt flow index (MFI) value. However, the negative impact of talc on impact strength can be reduced by using different types of fillers or fibres. Kodal et al. [8] studied the mechanical and physical properties of natural zeolite-filled PP and found that the hardness and density of zeolite-filled PP composites were higher than those of pure PP; these composites also showed higher yield strength, impact strength, and stiffness. Mokhtar et al. [9] found that the addition of polypropylene-grafted maleic anhydride and montmorillonite to PP/ABS composites resulted in higher strength and stiffness at the expense of toughness. Ayrilmisc et al. [10] studied the hardness, attenuation, and water resistance of PP composites with the addition of montmorillonite and almond shell powder. It was found that the water absorption decreased with an increase in the amount of montmorillonite, the hardness of the nonrotted composites was the highest, and the hardness of the decayed composites was the lowest. Kong et al. [15] mixed different contents of hexagonal boron nitride (hBN) with PP and found that when the hBN content was only 1%, the Young’s modulus value of the PP/hBN composite was 17.5% larger than that of pure PP, and the thermal conductivity achieved a 72.3% improvement.

However, to our knowledge, there have been few reports on the use of kaolin as a filler. Kaolin has the inorganic structure of Al_2_Si_2_O_5_(OH)_4_ and is a white clay that is naturally deposited in rocks. This material is characterised by a fine particle size, flaky structure, natural chemical inertness, nonflammability, and the potential to impart flame retardancy to polymer materials. Kaolin has been reported to improve the thermal stability, mechanical properties, and scratch resistance of PP [16]. Jikan et al. [17] observed that an increase in kaolin content increased the crystallisation temperature of PP, shortened the crystallisation time, and decreased the crystallisation activation energy. To enhance the mechanical and thermal properties of PP products, Yang et al. [18] prepared kaolin-filled PP composites and found that after adding kaolin, the thermal stability was enhanced, and the composites containing 3% kaolin demonstrated the best impact toughness, which was 30% higher than that of PP. The tensile strength and tensile strain of the composites also reached larger values. Hu et al. [19] prepared a kaolinite-based conductive powder (Sb-SnO2-kaolin) filled with PP to explore its electrical conductivity. The results showed that when the proportion of kaolinite-based conductive powder was 40%, the volume resistivity of Sb-SnO2-kaolin-PP composites was significantly reduced, which was 6~7 orders of magnitude lower than that of pure PP. Zhang et al. [20] introduced kaolin to enhance the flame retardancy of PP/intumescent flame retardant (IFR) composites, and the results showed that adding kaolin to the PP/IFR system could form a denser carbon layer, improve flame retardancy, and reduce smoke emission. The idea of combining layered minerals such as kaolin with polymers has several advantages. By varying the weight ratio of each reinforcement, a broad combination of mechanical properties can be achieved. There are economic advantages of replacing expensive reinforcements, such as glass fibres, with cheaper kaolin clays, and the mechanical properties of the composites can be enhanced. The mechanical anisotropy of injection samples can be reduced by replacing high-aspect-ratio fibre reinforcements, such as glass fibres, with layered mineral fillers such as kaolin [21].

As above, although it has been demonstrated that kaolin fillers improve the mechanical properties, thermal properties, flame retardancy, and electrical conductivity of polymer composites, the effect of kaolin on the microrheological properties of polymers has not yet been published. To date, only the microforming mechanisms of pure polymers [22,23,24,25,26,27,28], ceramic powders [29], and carbon fibre polymer composites [30] have been explored. Therefore, in this study, the microrheological properties of kaolin-filled PP (kaolin/PP) composites in high-aspect-ratio microrectangular grooves and their possible size effect were investigated. Here, we first prepared three kaolin/PP composites with different kaolin content weight ratios, measured the microviscosity value of the composites by using a capillary rheometer, and studied the microrheological properties of kaolin/PP composites by changing the melt temperature and shear rate. The influence of the kaolin content on the n of the non-Newton index and the K of the consistency coefficient was analysed. Finally, microinjection combined with ultrasonic-assisted demoulding was used to fabricate high-aspect-ratio microstructured prisms on kaolin/PP composites, and the relationship between processing parameters and filling rate and geometry shape replication was studied, which comprehensively revealed the microrheological phenomenon of kaolin/PP composites in high-aspect-ratio microinjection. In addition, the changing laws of friction and mechanical properties of kaolin/PP composites were studied. As a result, the optimal composite formulation of kaolin/PP composites coupled with a high-aspect-ratio microinjection moulding process was found. In conclusion, the purpose of this study was to develop a hybrid composite of kaolin in PP, not only to determine the effect of the kaolin content on the properties of the composites but also to investigate the high-aspect-ratio microinjection moulding mechanism of the composites. To the best of our knowledge, this is the first occurrence of high-aspect-ratio microinjection moulding of kaolin/PP composites, in which the microstructured samples have ideal profile replication and mechanical and friction properties. This investigation sheds light on future application prospects of microinjection moulding of kaolin/PP composites.

## 2. Results

### 2.1. Microviscosity of Kaolin/PP Composites

**Figure 1:**
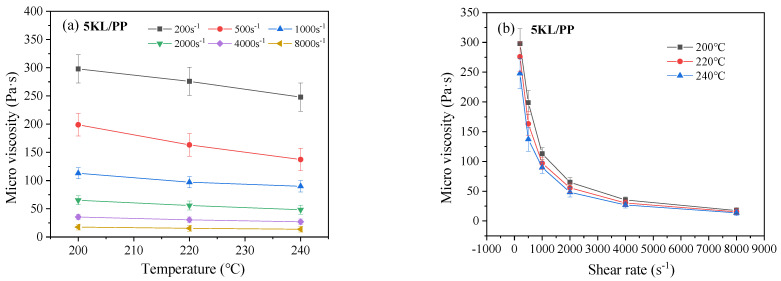
Microviscosities of the 5KL/PP composite at different (**a**) temperatures and (**b**) shear rates.

The microviscosity of kaolin/PP composites (5KL/PP) containing 5% kaolin at different temperatures and shear rates are shown in Figure 1. It was found that the microviscosity of the 5KL/PP composite decreased with increasing melt temperature and shear rate. At a low shear rate, the melt temperature of 5KL/PP has a significant effect on the microviscosity (Figure 1a). At this time, the microviscosity of the composite can be effectively reduced, and the flow properties of the material can be improved with increasing temperature. For example, when the shear rate is 200 s^−1^, the melt temperature increases from 200 °C to 240 °C, and the microviscosity value of the composite decreases by 50.1 Pa·s. However, at high shear rates, the effect of temperature decreases. For example, when the melt temperature increases from 200 °C to 240 °C at 4000 s^−1^, the microviscosity value only decreases by 3.7 Pa·s.

In addition, we can see from Figure 1b that with increasing shear rate, the microviscosity value decreases sharply at a low shear rate, and when the rate increases to 2000 s^−1^, the viscosity value decreases slowly. At the same time, the effect of the shear rate on the microviscosity of 5KL/PP composites is greater than that of temperature, indicating that the shear rate is more conducive to improving the fluid flow properties of the composites and reducing the microviscosity than the temperature.

**Figure 2:**
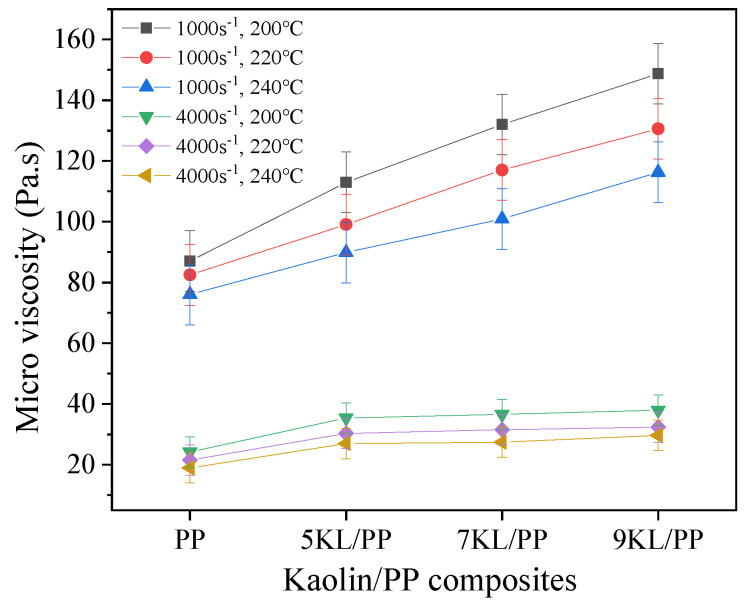
Effect of the kaolin content on the microviscosity of kaolin/PP composites.

To explore the effect of the change in kaolin content on the microviscosity of the kaolin/PP composites, we studied composite samples with 0%, 5%, 7%, and 9% kaolin contents at shear rates of 1000 s^−1^ and 4000 s^−1^ and temperatures of 200 °C, 220 °C, and 240 °C. A kaolin content of 0% means a pure PP material (Figure 2). It can be seen that the higher the kaolin content is, the higher the microviscosity of the composites. In addition, when the kaolin content was increased from 5% to 9%, the microviscosities of all melt temperatures increased by approximately 33 Pa·s at a shear rate of 1000 s^−1^, but at a shear rate of 4000 s^−1^, the microviscosities were basically unchanged. This shows that the higher the shear rate is, the kaolin content basically does not affect the microviscosity of the composites.

### 2.2. Microscopic Non-Newtonian Index

Since the melt flow of the composites after adding kaolin still behaves like a typical pseudoplastic fluid, at a low shear rate, the chain-like polymer chains are hooked and entangled with each other, and the viscosity is high. When the shear rate is high, due to the enhanced shear stress between the flow layers, the relatively scattered chain particles are rotated and contracted into clusters, which reduces the mutual hooking and the phenomenon of shear thinning occurs. The flow law conforms to the Power_Law power law flow model, so the Power_Law equation (Equation (1)) is used to fit the experimental data of the microviscosity:

(1) $$\eta =K\cdot {\overset{\cdot }{\gamma }}^{n-1}$$

where η is the microviscosity, Pa·s; $\dot{\gamma }$ is the shear rate, s^−1^; n is the microscopic non-Newtonian index; and *K* is the melt consistency.

**Figure 3:**
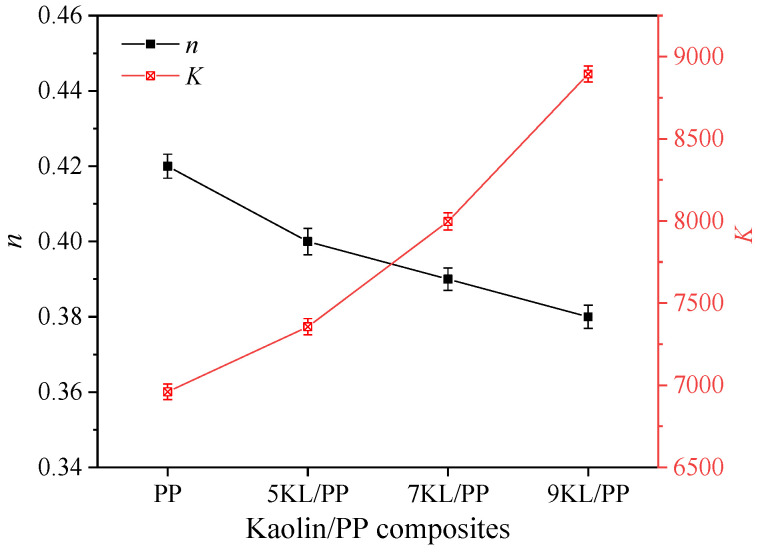
Microscopy non-Newton index and consistency coefficient of the kaolin/PP composites.

The microscopic non-Newtonian index n and melt consistency *K* of kaolin/PP composites with different kaolin contents were obtained (Figure 3). It can be seen that with increasing kaolin content, the microscopic non-Newton index n decreases linearly, and the consistency *K* value of the corresponding composite melt increases exponentially, indicating that the higher the kaolin content is, the more sensitive the kaolin/PP composites are to the shear rate, that is, the more obvious the shear thinning behaviour. This is because the matrix material PP itself is relatively non-Newtonian, and its branched chain structure makes the molecular chains difficult to entangle. As the kaolin content increases, the molecular chains are more difficult to entangle and the melt microscopic non-Newtonian index *n* decreases.

### 2.3. High-Aspect-Ratio Microgrooves

#### 2.3.1. Filling Rate

To study the microgroove filling properties of kaolin/PP composites, microinjection experiments were performed on kaolin/PP composites in this study. The microgroove filling rate was calculated by using Equation (2):

(2) $$F_{s}=\frac{H_{i}}{D_{m}}\times 100\%$$

where $H_{i}\;\mathrm{and}\;D_{m}$ are the height of the microstructured prismatic sample and the depth of the mould microgroove, respectively, and $F_{s}$ is the filling rate. The larger the value of $F_{s}$ is, the better the filling effect.

**Figure 4:**
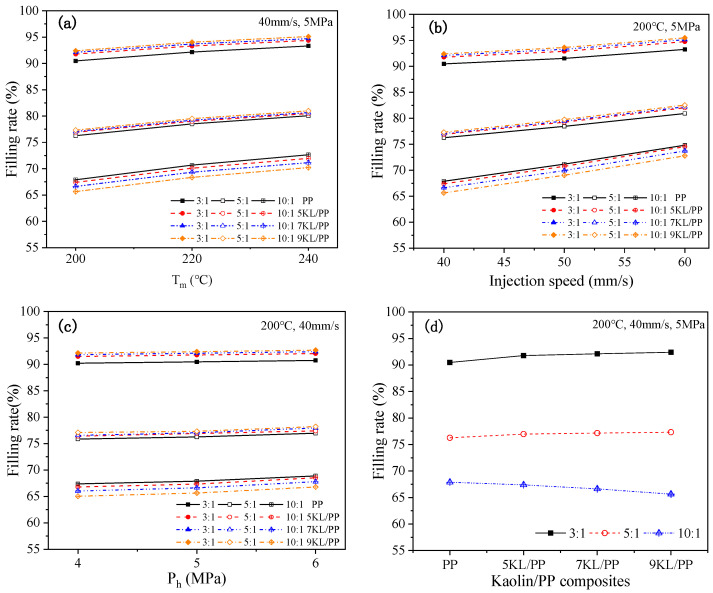
Effects of the (**a**) melt temperature, (**b**) injection speed, (**c**) hold pressure, and (**d**) kaolin content on the filling rate of microgrooves.

Figure 4 shows the change in the filling rate of PP and kaolin/PP composites in microinjection moulding in microgrooves with different aspect ratios under a single factor, that is, changing the melt temperature, injection speed, hold pressure, and kaolin content. For different aspect ratios, the filling rate of the rectangular microgrooves increases with increasing melt temperature under the conditions of an injection speed of 40 mm/s and a hold pressure of 5 MPa (Figure 4a). At the same time, the increase in the filling rate of the microgroove is nonlinear with an increasing melt temperature. For example, for a microgroove with an aspect ratio of 3:1, when the melt temperature was increased from 200 °C to 220 °C, the filling rate of the 7KL/PP composite increased from 92.11% to 93.69%, which is an increase of 1.72%. When the melt temperature was further increased, the filling rate of the 7KL/PP composite only increased by 1.05% under the same temperature increment of 20 °C (220 °C to 240 °C), and the increase slowed down.

Figure 4b shows the change in the filling rate of the PP and kaolin/PP composites at different injection speeds under the conditions of a melt temperature of 200 °C and a hold pressure of 5 MPa. The filling rate increases linearly with increasing injection speed.

For all microgrooves with different aspect ratios, the filling rate increases slightly with increasing hold pressure (Figure 4c). The hold pressure is an important process parameter in the microinjection moulding process. It is mainly used to solidify the melt under the action of pressure after injection moulding. Increasing the hold pressure can make more composite melt flow into the mould cavity, which is beneficial for the feeding and compaction inside the microgroove cavity, resulting in a compact sample, thereby improving the filling performance of the microgroove.

Figure 4d shows the change in the filling rate with different kaolin contents under different aspect ratio microgrooves and under the conditions of a melt temperature of 200 °C, an injection speed of 40 mm/s, and a hold pressure of 5 MPa. When the aspect ratio is small, that is, 3:1 and 5:1, the filling rate of the microgroove of the composites increases gradually with increasing kaolin content. However, when the aspect ratio is large, that is, 10:1, with increasing kaolin content, the filling rate of the microgrooves of the composites decreases gradually, and a size effect appears.

#### 2.3.2. Geometry

**Figure 5:**
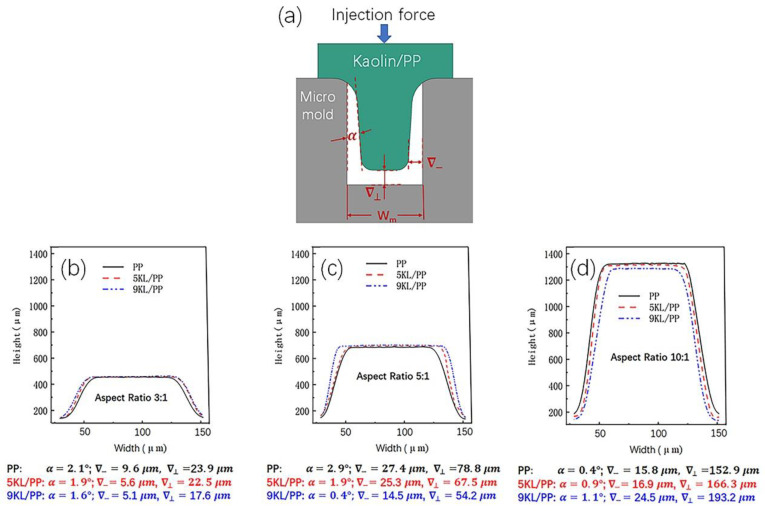
Geometry of the kaolin/PP composite (**a**) microstructured prismatic samples, with aspect ratios of (**b**) 3:1, (**c**) 5:1 and (**d**) 10:1.

In addition to the microgroove filling rate, the flow of the kaolin/PP composites in the microporous channel also determines the geometry of the microstructured prismatic samples and, thus, the dimensional accuracy of the samples. Here, according to the filling rate (Figure 4), we selected three typical materials, pure PP, 5KL/PP, and 9KL/PP, and analysed the geometries of the microstructured prismatic samples at an injection temperature of 240 °C, an injection speed of 40 mm/s and a hold pressure of 5 MPa under three different aspect ratios (Figure 5).

We randomly selected microstructured prismatic samples with every aspect ratio to study the morphological changes in the structure during the inflow of the kaolin/PP composites into the microgrooves. As shown in Figure 5a, the angle between the microstructured sample and the cavity wall of the mould is set as α, the lateral gap between the top of the microstructured sample and the mould is $\nabla_{-}$, and the axial gap with the mould is $\nabla_{⊥}$. Then, we extracted microstructure profiles with different aspect ratios from the LSCM results (Figure 5b–d). Using the same height scale, we can clearly find that the overall profile of the microstructured samples exhibits a trapezoidal platform shape in all cases. This is due to the shear deformation of the kaolin/PP composites on the surface of the mould during microinjection and the vertical axial deformation in the centre of the microgroove, resulting in the initial filling mode of a unimodal shape. With the continuous influx of the composites to flow, the clear single peak becomes inconspicuous, and then a trapezoidal flat surface is formed under the action of the cavity wall of the mould [31].

At the same time, it was found that when the aspect ratio was 3:1 and 5:1, with increasing kaolin content, the angle α gradually decreased, and the lateral gaps $\nabla_{-}$ and axial gaps $\nabla_{⊥}$ also gradually decreased. Especially for the 9KL/PP composites, α, $\nabla_{-}$, and $\nabla_{⊥}$ values are the smallest, and the shape reproduction is also the best. In addition, with increasing aspect ratio, all the angles (α) change slightly, but both the lateral gaps $\nabla_{-}$ and axial gaps $\nabla_{⊥}$ increase significantly. For example, when the aspect ratio of the 9KL/PP composite is 3:1, the lateral gap is 5.1 μm and the axial gap is 17.6 μm. However, when the aspect ratio is 5:1, the lateral gap is 14.5 μm and the axial gap is 54.2 μm; these values are approximately three times larger than those when the aspect ratio is 3:1.

However, when the aspect ratio is 10:1, with increasing the kaolin content, the change rules of α, $\nabla_{-}$, and $\nabla_{⊥}$ are opposite to the previous, showing a gradually increasing trend, and a size effect appears. At this time, the shape reproduction of pure PP material is the best.

### 2.4. Mechanical Properties of the Kaolin/PP Composites

#### 2.4.1. Friction Performance

**Figure 6:**
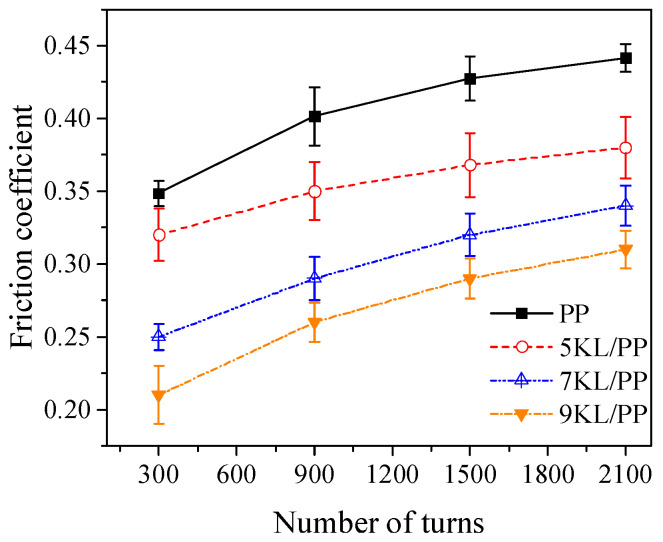
Relationship between the friction coefficient and numbers of turns and kaolin content of kaolin/PP composites.

Figure 6 shows the variation in the friction coefficient of the kaolin/PP composites with the number of turns and kaolin content. The friction coefficient of pure PP and kaolin/PP composites increases with increasing number of turns. However, regardless of the number of turns, the friction coefficients of the kaolin/PP composites are all smaller than that of pure PP. With increasing kaolin content, the average friction coefficient of the composites at different number of turns also decreased accordingly.

#### 2.4.2. Mechanical Properties

**Figure 7:**
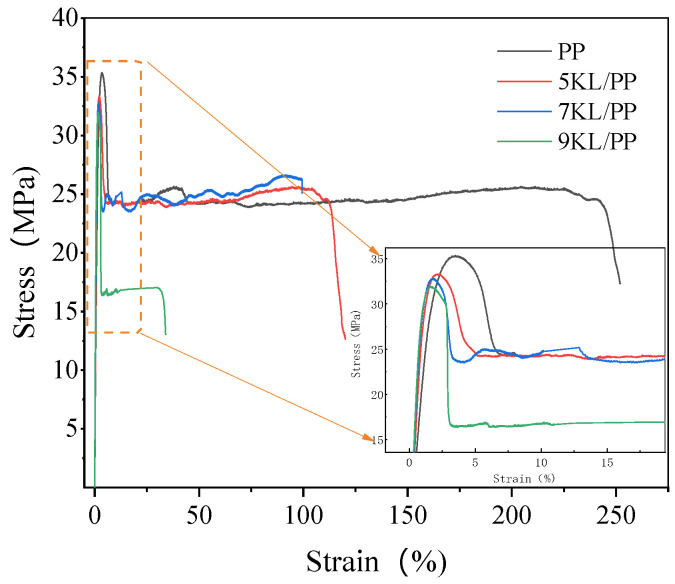
Stress–strain curve of the kaolin/PP composites.

The typical stress–strain curve of kaolin/PP composites is shown in Figure 7. To maintain consistency with the microinjection process, the set of process parameters in Table 1 was randomly selected. The injection temperature, injection speed, and hold pressure of the tensile sample were 240 °C, 40 mm/s, and 5 MPa, respectively.

We found that when the load was applied to the kaolin/PP composites, the stress started to increase sharply and was in the stage of elastic deformation. However, after reaching the maximum stress, the stress decreased sharply and then entered a stable plastic deformation stage under a certain stress until the sample could not bear the load and ductile fracture occurred.

**Figure 8:**
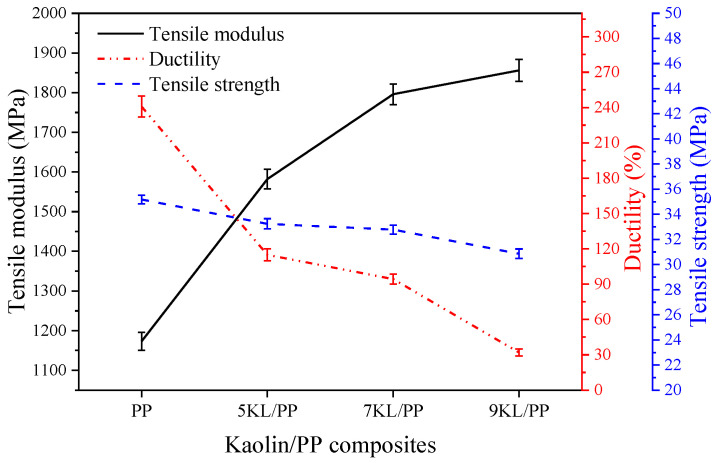
Mechanical properties of PP/kaolin composites.

In Figure 8, it can be observed that after adding kaolin filler, the tensile strength of the composites decreased slightly. Compared with the pure PP material, the strength of the 5KL/PP and 7KL/PP composites is only reduced by approximately 5.5% and 6.8%, respectively. However, the elastic modulus is increased significantly, increasing by 34.8% and 53.1%. This shows that the kaolin/PP composite has a significant enhancement effect in terms of tensile modulus and stiffness under the condition of small strength loss, which is beneficial for improving the practicability of the composites.

## 3. Discussion

### 3.1. Microviscosity Analysis

The effect of the melt temperature on the microviscosity is because when the melt temperature of the composites is low, the polymer PP molecular chain of the composite matrix presents an entangled state, the gap between the molecular chains is small, and the melt flow resistance increases. As the temperature increases, the molecular activity increases, the free volume of the material is increased, the molecular chain spacing increases, the molecular entanglement state decreases, the flow resistance decreases, and the melt viscosity decreases. When the shear rate is high, the shear stress can effectively unwind the entangled state of the molecular chain, and the molecular chain is opened, that is, the phenomenon of shear thinning [27,32]. The melt flow is effectively improved, and the viscosity is reduced.

**Figure 9:**
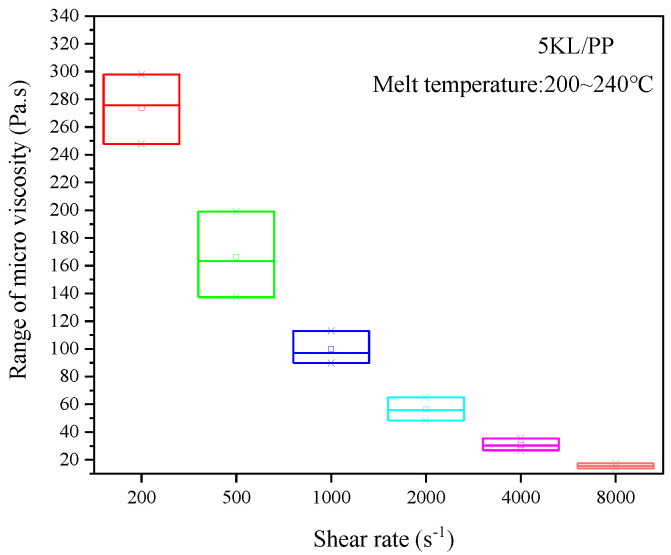
Effect of temperatures on the microviscosity under different shear rates in the form of standard deviation.

Figure 9 shows the effect of temperatures on the microviscosity under different shear rates in the form of standard deviation. The wider the range of the box plot, the larger the standard deviation, indicating that the fluctuation of the microviscosity value is larger; therefore, the influence of temperatures on the microviscosity is greater. It could be seen that when the shear rate is small, the temperature has a great influence on the microviscosity; and when the shear rate is greater than 4000 s^−1^, the temperature has basically no effect. It shows that the increase in shear rate weakens the effect of temperature on microviscosity, and shear rate has a greater effect on microviscosity than temperature.

Furthermore, with the increase of kaolin content, the microviscosity of kaolin/PP composites increases (Figure 2), which may be mainly due to two reasons. First, the fluidity of kaolin itself is worse than that of the PP matrix. With increasing kaolin content, the fluidity of the composites is affected, resulting in a continuous increase in the viscosity of the composites. The other is that after adding kaolin, kaolin is dispersed between polymer molecular chains, which reduces the interfacial voids between clay and polymer molecular chains. At the same time, the sheet rigid structure of kaolin hinders the flow of molecular chains. The interaction of kaolin and PP increases the flow resistance of the composite melt, and the microviscosity value of the composites increases significantly.

**Figure 10:**
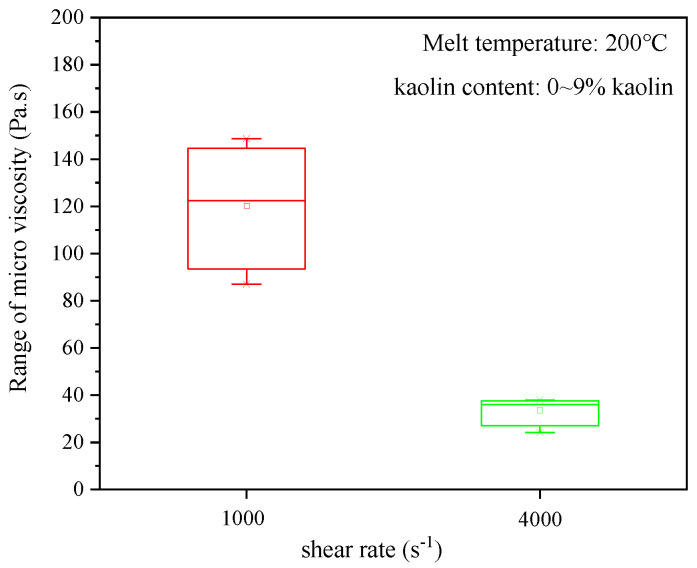
Effect of kaolin content on the microviscosity in the form of standard deviation.

At the same time, it can be seen from Figure 10 that the kaolin content has a greater influence on the microviscosity at the low shear rate of 1000 s^−1^ than that at a high shear rate of 4000 s^−1^. The kaolin content has little effect on the microviscosity at high shear rates. The increase in shear rate also weakens the effect of kaolin content on microviscosity.

In short, although the microviscosity of kaolin/PP composites is affected by the kaolin content, shear rate, and melt temperature, the shear rate has the greatest effect, especially at high shear rates.

### 3.2. Filling Rate Analysis

The filling rate of the rectangular microgrooves increases with increasing melt temperature (Figure 4a). This is mainly caused by three factors. One is that increasing the melt temperature can reduce the melt viscosity of kaolin/PP composites (Figure 1a), which can effectively improve the flow properties of the melt. After the melt temperature is increased, the condensation layer formed on the mould wall when the composite melt fills the mould cavity decreases, which reduces the flow resistance when filling the microgrooves. Third, increasing the melt temperature reduces the cooling rate, which can effectively improve the effect of subsequent hold pressure, and improve the filling rate of the microgrooves.

At the same time, the increase in the filling rate of the microgroove is nonlinear with increasing melt temperature. This may be because the composite is a non-Newtonian fluid, the melt temperature increases, the melt volume expands, and the melt volume entering the mould cavity decreases, resulting in a decrease in the increase in the microgroove filling rate.

The influence of the injection speed on the filling rate of the microgroove is mainly reflected in the following two aspects. One aspect is that the increase in the injection speed can increase the shear rate of the composite melt so that the shear heat generation effect inside the mould cavity during the microinjection process is enhanced. The melt temperature is further increased, which effectively improves the fluid flow properties, reduces the melt viscosity, and facilitates microgroove filling. The other aspect is that the increase in the injection speed means that the time for the melt to fill the microgroove is reduced, thereby reducing the contact time between the composite melt and the mould wall. The reduced contact time is beneficial for slowing down the formation of the condensation layer inside the mould cavity, which is beneficial for the feeding process, and more composite melt will flow into the mould cavity. Additionally, the filling rate of the microgroove is also increased.

**Figure 11:**
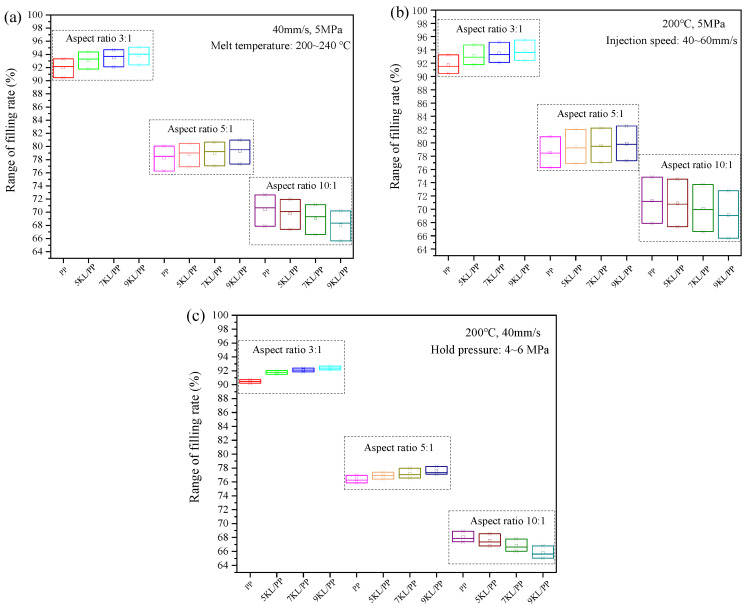
Effect of (**a**) melt temperature, (**b**) injection speed, and (**c**) hold pressure on the filling rate at different kaolin content and aspect ratios in the form of standard deviation.

It can be seen from Figure 11 that under different aspect ratios, the influence of kaolin content on the filling rate of microgrooves is different. When the aspect ratio is small, namely 3:1 and 5:1, the filling rate of microgrooves increases with the increase of kaolin content regardless of the melt temperature, injection speed, and hold pressure. When the aspect ratio increases 10:1, the filling rate decreases with the increase of kaolin content.

We speculate that different factors control the composite flow. When the aspect ratio is small, although the melt viscosity of the composites increases after adding kaolin, the resistance of the melt flowing into the microgroove with a small aspect ratio is small, and the influence of viscosity can be ignored. Second, since kaolin is a flaky mineral product, it is a rigid particle, and its shrinkage performance is smaller than that of PP itself. In addition, the orientation of PP crystallites in kaolin/PP composites is much lower than that of pure PP, which also leads to a decrease in shrinkage [11]. Third, after adding kaolin, the interior of the plastic sample is supported so that the polymer molecular chain is well supported, reducing the cantilever and dangling effect of the molecular chain, improving the rigidity of the composites, and reducing the moulding shrinkage rate. At the same time, as the kaolin content increases, the microscopic non-Newtonian index of the melt decreases (Figure 3), the melt is more sensitive to the shear rate, the shear thinning behaviour is more obvious, and the melt filling performance is better. In short, the microgroove filling rate can be effectively improved due to the reduced shrinkage rate of the composites, while the influence of melt viscosity can be ignored at this time.

However, when the aspect ratio reaches 10:1, due to the increased contact time between the composite melt and the mould wall in the microgroove, the heat exchange is intensified, the melt is rapidly cooled, and the viscosity increases, making the melt flow resistance increase when the melt fills the microgroove. At the same time, the addition of kaolin increases the melt viscosity of the composites (Figure 2), which hinders the filling of the microgroove. This is consistent with the findings of Trotta et al. [28], who believed that under extreme conditions of high aspect ratio, viscosity is the most important factor for melt flow, and low-viscosity materials are more conducive to melt flow and obtain a larger filling rate.

In summary, in the microinjection experiment, when the aspect ratio is small, the increase in melt viscosity caused by kaolin has little effect on the filling of the microgrooves, but the shrinkage reduction of the composites caused by kaolin as a filler has a greater impact on the filling performance. At this time, the microgroove filling rate increases with increasing kaolin content. As the aspect ratio increases, the influence of kaolin on reducing shrinkage of the composite is weakened. The most influential factor is the viscosity value of the composites themselves, so the higher the kaolin content, the lower the microgroove filling rate.

At the same time, we also noticed that under the conditions for all aspect ratios and any kaolin content, the range of the filling rate box plot caused by the change of injection speed is the widest (Figure 11b), and the range of the filling rate box plot caused by the change of hold pressure is the narrowest (Figure 11c). It indicates that the injection speed has the greatest impact on the filling rate, followed by the melt temperature, and the hold pressure has the least impact.

### 3.3. Geometry Analysis

For the influence of aspect ratios on the geometry of the microstructured prismatic samples, we speculate that different factors control the flow morphology of composites with different aspect ratios, including viscosity-related flow properties, such as time dependence, temperature, and kaolin content.

**Figure 12:**
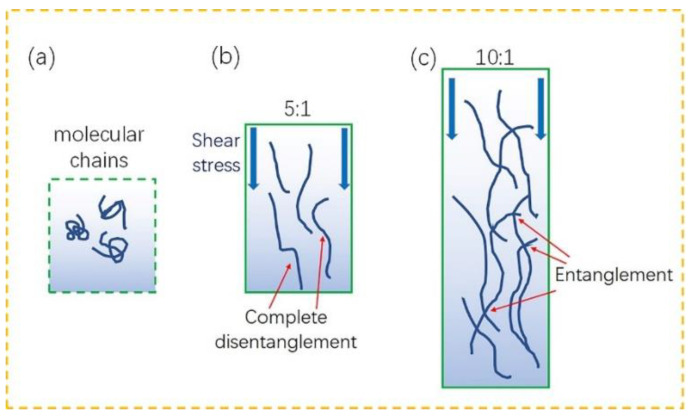
Behaviour of kaolin/PP composites: (**a**) molecular chains under shear stress with aspect ratios of (**b**) 5:1 and (**c**) 10:1.

**Figure 13:**
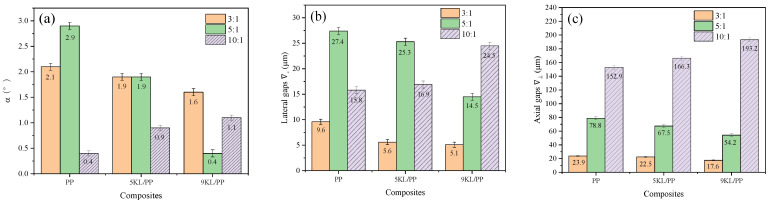
Effect of different aspect ratios and different composites on (**a**) the angle α, (**b**) the lateral gaps $\nabla_{-}$, and (**c**) the axial gaps $\nabla_{⊥}$.

During microinjection, the viscous flow and shear deformation work together on the flow of the composites [32]. In the microgroove with a small aspect ratio, the shear deformation has a great influence, which dominates the flow of the composites. As a result, the 9KL/PP composite with high viscosity exhibits an obvious shear thinning phenomenon near the surface of the mould cavity under the action of shear deformation. Therefore, the molecular chains of the kaolin/PP composites can be gradually disentangled, and the melt fluidity is improved (Figure 12b). The molecular chain of the central part is also gradually disentangled under the action of shear stress, so it can also flow quickly, which is why the angle α, the lateral gap $\nabla_{-}$, and the axial gap $\nabla_{⊥}$ of the 9KL/PP composite are small (Figure 13).

Second, in the microgroove with a smaller aspect ratio, the higher the viscosity is, the more obvious the shear thinning phenomenon of the kaolin/PP composites [27]. This is because the higher the kaolin content is, the larger the distance between the molecular chains of the composites and the less molecular entanglement, which enables the composites under the action of shear stress to more easily flow, and the better the geometric shape of the microstructured sample replication effect. On the other hand, when the kaolin content is high, the kaolin additive acts as a skeleton, reducing the shrinkage of the composites and improving their replication ability.

When the microgrooves have a larger aspect ratio (10:1), the composite flow is less affected by shear deformation. This is because under extreme conditions, such as an excessively large aspect ratio, the molecular chains of the composites may suffer from shear stress. However, because the depth of the microgrooves is too large, the unwrapped molecular chains are entangled with each other again, which weakens the molecular mobility (Figure 12c). At this time, the shear effect near the cavity wall is reduced, and the shear thinning phenomenon is weakened. The axial deformation of the central part mainly depends on the viscosity of the composites themselves. The smaller the viscosity is, the better the flow of the composites. The viscous flow of the composites along the axial direction determines the final microstructured prismatic sample geometry of the kaolin/PP composites, resulting in the best geometric replication for the smallest viscous PP material. It can be seen from Figure 13 that in the microgrooves with an aspect ratio of 10:1, the pure PP material has the smallest angle α, lateral clearance $\nabla_{-}$, and axial clearance $\nabla_{⊥}$, so it has the best geometric replication effect.

We tend to believe that in low-aspect-ratio microgrooves, shear deformation dominates the flow of kaolin/PP composites, while in high-aspect-ratio microgrooves, the viscosity of the material itself dominates the composite flow.

In addition, with increasing aspect ratio, the axial gap $\nabla_{⊥}$ of all composites increases significantly (Figure 13c). Apart from the viscosity, it is also possible that the air in the mould cavity hinders composite flow. The greater the aspect ratio is, the greater the air resistance.

### 3.4. Mechanical Properties Analysis

The average friction coefficient of the composites decreased with increasing kaolin content, which is because the friction contact surface of pure PP has a larger contact area, which requires a larger friction force, resulting in a higher friction coefficient when the material is rubbed. For kaolin/PP composites, due to the flaky rigid structure of the silicate layer on the surface of the sample, kaolin reduces the real contact area of the frictional contact surface of the sample and reduces the friction during the friction process. On the other hand, for the PP/kaolin composites with the addition of hard kaolin powder, the wear mechanism may coexist with abrasive wear and adhesive wear, thereby reducing the friction coefficient of the composites. The small friction coefficient can reduce the loss of the composites and the heat generated in the friction process, prevent local overheating, and improve the wear resistance of the composites.

**Figure 14:**
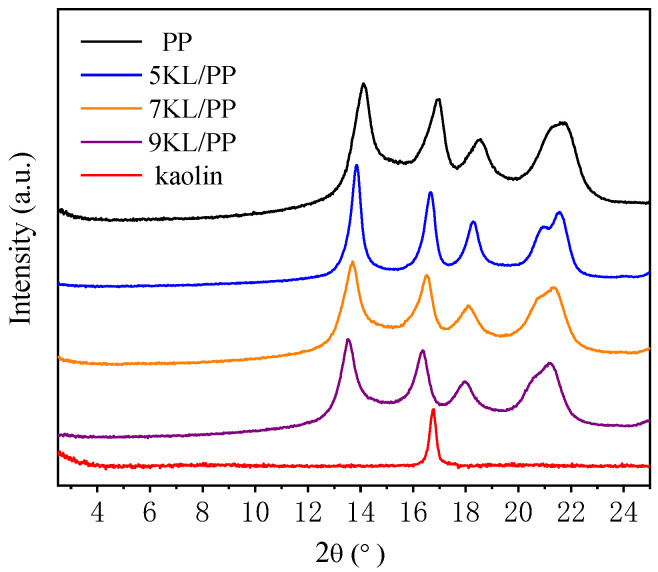
X-ray diffraction patterns.

The significant increase in the elastic modulus of the kaolin/PP composites is mainly due to the intercalation effect of kaolin. The XRD patterns of kaolin/PP composites with different formulations are shown in Figure 14.

The characteristic peak (001) of pure kaolin (red line) is observed around 16.6°. According to the Bragg equation, the interlayer spacing d = nλ/2sinθ, where *n* = 1, λ = 0.15406 nm, and θ is the angle corresponding to the diffraction peak. Then, the calculated interlayer spacing of pure kaolin is 0.531 nm. The XRD pattern of pure PP polymer (black line) exhibits four characteristic peaks. When kaolin is packed into PP polymer, its four characteristic peaks move slightly to lower angles, which demonstrates the presence of the effect of kaolin dispersed in the polymer matrix.

At the same time, the first diffraction peaks of the kaolin/PP composites are all shifted to lower angles compared with the diffraction peaks of pure kaolin; for example, for the 9KL/PP composite, the (001) diffraction peak shifts from 16.6° to 13.76°. Therefore, the kaolin interlayer spacing increases from 0.531 nm to 0.643 nm during melt blending. This result clearly shows that PP macromolecular chains have been inserted between the clay layers. The increase in the kaolin interlayer spacing may be due to the organic surfactant increasing the interlayer distance and reducing the electrostatic attraction between adjacent lamellae, thus providing the possibility for the diffusion of PP molecular chains between the kaolin layers during processing [23].

In addition, with increasing kaolin content, the interlayer spacing of kaolin/PP composites increases from 0.636 to 0.643 nm, which is more conducive to the polymer intercalation effect. Moreover, the planar bonding between the kaolin clay sheet and the PP polymer is enhanced, which is helpful for improving the stiffness of composites.

**Figure 15:**
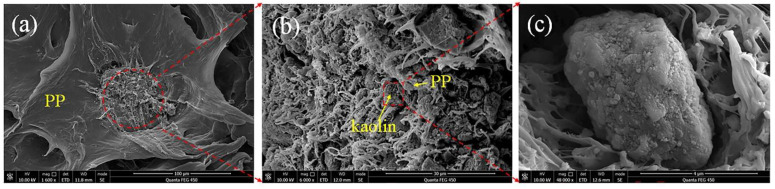
(**a**) ESEM image of fracture of 9KL/PP composite, (**b**) the aggregated kaolin region in (**a**), and (**c**) the kaolin in (**b**).

Generally, the strength of the kaolin/PP composites should increase due to kaolin powder filling; however, Figure 9 shows that the strength decreases slightly. The reason may be due to the uneven distribution of kaolin clay in the PP matrix. With increasing clay content, the aggregation of clay in the polymer matrix is more obvious (Figure 15). The poor dispersion results in a gradual decrease in strength. On the other hand, due to the strong and hard rigid structure of kaolin clay itself, it has a strong strength. Although its dispersibility is poor, due to the particle strengthening effect [33], the overall effect of kaolin filling on the strength of the composite is less.

Furthermore, it can be seen (Figure 8) that the ductility of pure PP and the 5KL/PP composite are 240.9% and 117.94%, respectively. The ductility of 5KL/PP is 51.1% lower than that of PP. The ductility of the composites decreases significantly; particularly, when the kaolin clay content is increased to 9%, the ductility decreases sharply. This is also due to the aggregation of kaolin in the PP matrix, resulting in poor dispersion. Figure 15a is the fracture of 9KL/PP composite. Although kaolin is dispersed in the PP matrix, it is obvious that the kaolin aggregates together to form a region with a diameter of approximately 70 μm. Moreover, in the aggregated region, the kaolin powder is dispersed by the PP matrix again (Figure 15b). At the same time, it was also found that the kaolin powders have the plate-like pseudohexagonal shape of the original kaolinite (Figure 15c). Mechanical activation decreases the particle size [34], and the size of kaolin particle is reduced from the original 10 μm to about 4 μm. Although the size of kaolin particle decreases after mechanical activation, the interfacial adhesion stress between the unevenly dispersed kaolin particles and the PP matrix increases. Therefore, after the tensile deformation of the sample, with increasing plasticity, the local stress increase causes cracks to occur and expand, resulting in a decrease in the ductility.

In summary, it can be seen that the 7KL/PP composite exhibits good elastic modulus with relatively small loss of strength and ductility (Figure 8), and it has good filling properties and geometric shape replication effect (Figure 5) when microinjected with a small aspect ratio. The wear resistance of the microstructured samples is also good (Figure 6). This shows that the 7KL/PP composite samples have excellent comprehensive properties.

In addition, compared with the conventional PP composites containing 7% carbon fibre [35,36], the elastic modulus of 7KL/PP increased by 12.5%, and the tensile strength was basically the same. Although the microviscosity of 7KL/PP is slightly higher, due to its smaller shrinkage, the microstructure replication accuracy of its small aspect ratio is higher. Moreover, the price of kaolin is much lower than that of carbon fibre, so it has broad application prospects to develop low-cost silicate polymer composites and improve the moulding accuracy and mechanical properties of their microparts.

## 4. Materials and Experiments

### 4.1. Materials

The main materials in the experiment are polypropylene particles (PP, 9020M, molecular weight 80,000, and density 0.9 g/cm^3^, Sinopec Maoming Branch, Maoming, China) and kaolin powder (kaolin, particle size 10 μm, pore volume 0.2 cm^3^/g, and specific surface area 17 m^2^/g, Tianjin Zhiyuan Chemical Reagent Co., Ltd., Tianjin, China). First, PP particles and kaolin powder were placed in a vacuum drying oven (DZ-6050B, Wohong Experimental Instrument Co., Ltd., Jinan, China) and dried at a temperature of 80 °C for 4 h. After drying, PP and kaolin were weighed in a certain proportion, and then the PP and kaolin were placed into a horizontal planetary mixer (MSK-SFM-1, Hefei Kejing Material Technology Co., Ltd., Hefei, China) and mixed for 20 min. At 300 r/min, three different proportions of kaolin/PP composites with kaolin weight contents of 5%, 7%, and 9% were prepared. For all formulations, 1% maleic anhydride grafted PP was added to strengthen the combination of kaolin and PP matrix material. In this paper, the kaolin/PP composite samples are referred to as KL/PP; for example, for the 5KL/PP sample, 5 represents 5% kaolin, KL represents kaolin, and so on.

### 4.2. Experiments

#### 4.2.1. Microviscosity Experiment

A double-barrel capillary rheometer (RH7, NETZSCH Company, Selb, Bavaria, Germany) was used. During the experiment, after heating the barrel to the set temperature, the material bar above the barrel extruded the kaolin/PP composite melt from the die at the set speed. At this time, the pressure sensor measured the pressure. The relationship between the melt microviscosity and shear rate of the kaolin/PP composites was calculated and output to the display.

To reflect the microviscosity value of the kaolin/PP composite melt, we chose a capillary die with a diameter of 1 mm, a length of 16 mm, and an aspect ratio of 1:16. The tested shear rate values were 200 s^−1^, 500 s^−1^, 1000 s^−1^, 2000 s^−1^, 4000 s^−1^, and 8000 s^−1^, and the tested temperatures were 200 °C, 220 °C, and 240 °C, respectively. During the measurement, three repeated experiments were carried out under each condition, and the microviscosity value of the kaolin/PP composites was taken as the average value of the three experiments.

#### 4.2.2. Microinjection Experiment

**Figure 16:**
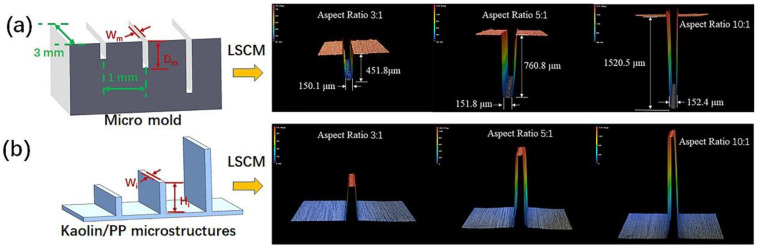
Cross-sectional dimensions of (**a**) microgrooves and (**b**) microstructured prismatic samples with aspect ratios of 3:1, 5:1, and 10:1.

The microinjection machine (model BABYPLAST-6-10P) was produced by Babyplast Company in Italy. The maximum injection volume was 15 cm^3^, and the maximum injection pressure and injection speed were 15 MPa and 80 mm/s, respectively. In this experiment, the microinjection moulds were designed with three different depths. Rectangular microgrooves with aspect ratios of 3:1, 5:1, and 10:1 were produced; each microgroove had a length of 3 mm, a spacing of 1 mm, a cross-sectional width W_m_, and a depth D_m_ (Figure 16a). The rectangular microgrooves of moulds were cut and formed by a slow wire feeder (Sodick AP250Ls, Japan Sodick Company, Fukui, Japan), and the cross section of the microgrooves was measured by laser scanning confocal microscopy (LSCM, VK-250, Keyence Company, Osaka, Japan).

To quantitatively characterise the replication performance of the rectangular microstructure, the microstructured prismatic samples were also measured by LSCM five times, and the average value was taken as the experimental result.

In addition to considering the aspect ratio of the microgroove, the microinjection moulding process parameters also have a great influence on the filling and geometric replication properties of the composite melt. The process parameters considered in this paper include the melt temperature (T_m_), injection velocity (V_i_), and hold pressure (P_h_). Three levels were selected for each process parameter, namely T_m_ was 200, 220, and 240 °C; V_i_ was 40, 50, and 60 mm/s; and P_h_ was 4, 5, and 6 MPa. The single factor method was used to study the influence of various factors on the filling and geometric replication performance [37]. The specific arrangement is shown in Table 1. The principle of experimental design is as follows. N1 in Table 1 is selected as the standard reference condition group. On the basis of N1, V_i_ and P_h_ are unchanged in the N2 and N3 groups, only T_m_ is changed; T_m_ and P_h_ are unchanged in the N4 and N5 groups, only V_i_ is changed; and T_m_ and V_i_ are unchanged in the N6 and N7 groups, only P_h_ is changed. Thereby, the number of tests was reduced, and the influence of each parameter was obtained quickly. In addition, during the experiment, other injection moulding process parameters remained unchanged; that is, the mould temperature was 25 °C, the injection pressure was 8 MPa, the injection time was 2 s, the pressure holding time was 4 s, and the cooling time was 12 s. Under each experimental condition, the first 10 samples under each process parameter were discarded, and after the machine was stable, five moulded samples were selected for laser confocal inspection to analyse the microgroove filling rate and sample geometric shape replication effect.

#### 4.2.3. Properties Test

The friction properties of the microstructured prismatic samples were tested using a homemade pin-on-disk tribometer and a nanoprobe displacement sensor [38]. Due to the limitations of experimental conditions, we only tested samples with an aspect ratio of 10:1. The friction head is a SiC ball (radius of 1 mm). In this study, the tribotests were performed at a 23.5 °C room temperature with a relative humidity of approximately 57% under a normal load of 2.0 N and a rotational speed of 180 rpm with a frictional radius of 0.45 mm.

The mechanical properties of the samples were tested by an automatic universal tensile testing machine (Z050TEW, Zwick Company, Ennepeta, Germany). To compare and analyse the microstructured prismatic samples, the injection moulding process of the tensile sample was completely consistent with that of the microinjection sample (Table 1), and the ISO 527-2:1993 1BA standard sample was selected for testing. The test speed was 5 mm/min, the maximum load applied was 50 kN, and all measurements were conducted at room temperature. To ensure reliable experimental results, five sample experiments were carried out on the tensile samples of each composite, the average value of the five results was taken as the experimental value, and the relative errors were calculated and counted.

To observe the interlayer changes in kaolin, we performed X-ray diffraction (XRD) experiments on the composites. In the experiment, an X-ray diffractometer (MiniFlex600, Rigaku Company, Tokyo, Japan) was used to detect the tensile samples of the kaolin/PP composites. The speed was 4 °/min, and the target material was Cu. Then, Jade6.0 software was used to process the measured data.

The fracture morphology of the tensile samples was observed with an environmental scanning electron microscope (ESEM, Quanta450FEG, FEI Company, Hillsboro, FL, USA). First, the surface to be observed was sprayed with gold, and then the samples were placed in a scanning electron microscope for detection.

## 5. Conclusions

In this study, the microrheological morphology of kaolin/PP composites with high-aspect-ratio microgroove injection moulding and the effects of kaolin clay contents on the mechanical properties were investigated. The microrheological properties of all formulated composites were analysed, as well as the microgroove filling rate and the sample geometry replication effect at three different aspect ratios. The coefficient of friction, tensile strength, tensile modulus, and ductility of the kaolin/PP composites were determined.

It was concluded that with increasing kaolin content, the microviscosity of kaolin/PP composites is gradually increased. The shear rate exhibits the greatest influence on the microviscosity, and the kaolin content demonstrates the least influence. This is because the fluidity of kaolin itself is worse than that of the PP matrix. With increasing kaolin content, the fluidity of the composites is affected, resulting in a continuous increase in the viscosity of the composites. Moreover, at the same time, kaolin is dispersed between polymer molecular chains, which reduces the interfacial voids between clay and polymer molecular chains. The interaction of kaolin and PP increases the flow resistance of the composite melt, and the microviscosity value of the composites increases significantly. When the shear rate is high, the shear stress can effectively unwind the entangled state of the molecular chain so that the viscosity is sharply reduced.

When the aspect ratio of microgrooves is 3:1 and 5:1, with increasing kaolin content, the microgroove filling rate increases, and the microstructured sample geometric shape replication effect is good. Shear deformation dominates the flow of the composites, and kaolin as a filler reduces material shrinkage and affects its filling properties. However, when the aspect ratio reaches 10:1, the microgroove filling rate decreases with increasing kaolin content, the microstructured sample geometric shape replication effect is also poor, and a size effect appears. At the same time, the viscosity of the composites itself controls the flow.

The intercalation effect and particle strengthening effect of the composites lead to a sharp increase in the elastic modulus, reaching 1856 MPa, and a slight decrease in the tensile strength. The reduction in the contact areas of the sample friction surface and the particle wear leads to a decrease in the friction coefficient and an increase in the wear resistance of the kaolin/PP composites.

Finally, we concluded that the best composite formulation for coupling kaolin/PP composites is the 7KL/PP composite with 7% kaolin. When the aspect ratio is 5:1, the microgroove filling rate is the highest, the reproduction of the microstructured sample geometry is the best, and the comprehensive mechanical properties of the sample are the best.

We believe that this work will contribute to many applications of silicate polymer composite microparts, such as improving micropart moulding accuracy and mechanical properties while simultaneously reducing economic costs.

## Figures and Tables

**Table 1:** Microinjection process parameters.

Number	T_m_ (°C)	V_i_ (mm/s)	P_h_ (MPa)
N1	200	40	5
N2	220	40	5
N3	240	40	5
N4	200	50	5
N5	200	60	5
N6	200	40	4
N7	200	40	6

## Data Availability

The data presented in this study are available on request from the corresponding author.
